# Electrospun Nanosystems Based on PHBV and ZnO for Ecological Food Packaging

**DOI:** 10.3390/polym13132123

**Published:** 2021-06-28

**Authors:** Maria Râpă, Maria Stefan, Paula Adriana Popa, Dana Toloman, Cristian Leostean, Gheorghe Borodi, Dan Cristian Vodnar, Magdalena Wrona, Jesús Salafranca, Cristina Nerín, Daniel Gabriel Barta, Maria Suciu, Cristian Predescu, Ecaterina Matei

**Affiliations:** 1 Faculty of Materials Science and Engineering, University Politehnica of Bucharest, 313 Splaiul Independentei, 060042 Bucharest, Romania; rapa_m2002@yahoo.com (M.R.); cpredescu56@yahoo.com (C.P.); 2 National Institute for Research and Development of Isotopic and Molecular Technologies, Donat Street 67-103, 400293 Cluj-Napoca, Romania; popa@itim-cj.ro (P.A.P.); dana.toloman@itim-cj.ro (D.T.); cleostean@itim-cj.ro (C.L.); gheorghe.borodi@itim-cj.ro (G.B.); maria.suciu@itim-cj.ro (M.S.); 3 Department of Food Science, University of Agricultural Sciences and Veterinary Medicine, 3-5 Calea Manastur, 400372 Cluj-Napoca, Romania; dan.vodnar@usamvcluj.ro (D.C.V.); gabriel.barta@usamvcluj.ro (D.G.B.); 4 Department of Analytical Chemistry, Aragon Institute of Engineering Research I3A, EINA-University of Zaragoza, Torres Quevedo Building, María de Luna 3, 50018 Zaragoza, Spain; magdalenka.wrona@gmail.com (M.W.); fjsl@unizar.es (J.S.); cnerin@unizar.es (C.N.)

**Keywords:** PHBV, Fe-doped ZnO nanoparticles, electrospinning, PLA, migration, antimicrobial, compositional analysis, food packaging

## Abstract

The electrospun nanosystems containing poly(3-hydroxybutyrate-co-3-hydroxyvalerate) (PHBV) and 1 wt% Fe doped ZnO nanoparticles (NPs) (with the content of dopant in the range of 0–1 wt% Fe) deposited onto polylactic acid (PLA) film were prepared for food packaging application. They were investigated by scanning electron microscopy (SEM), energy dispersive X-ray (EDX), Fourier transform infrared spectroscopy (FT-IR), X-ray diffraction (XRD), antimicrobial analysis, and X-ray photoelectron spectrometry (XPS) techniques. Migration studies conducted in acetic acid 3% (*wt*/*wt*) and ethanol 10% (*v*/*v*) food simulants as well as by the use of treated ashes with 3% HNO_3_ solution reveal that the migration of Zn and Fe falls into the specific limits imposed by the legislation in force. Results indicated that the PLA/PHBV/ZnO:Fex electrospun nanosystems exhibit excellent antibacterial activity against the *Pseudomonas aeruginosa* (ATCC-27853) due to the generation of a larger amount of perhydroxyl (˙OOH) radicals as assessed using electron paramagnetic resonance (EPR) spectroscopy coupled with a spin trapping method.

## 1. Introduction

Food packaging materials are essential in our economy and daily lives. The growing consumption of single-use food packaging includes trays, cups, bottles, bags, lids, straws, and cutlery, thus increasing the amount of plastic waste generated each year. Manufactured food packaging from conventional plastics like polyethylene (PE), polystyrene (PS), polypropylene (PP), and polyethylene terephthalate (PET) are a source of unintentionally produced microplastics (plastic fragments lower than 5 mm in size), generating a toxic effect both for the natural environment and human health [1]. Recent studies reported microplastics in air, drinking water, consumed milk, and other beverages [2]. A study conducted by Kedzierski et al. [3] reported that one person could be ingesting 1.4 mg of extruded polystyrene (XPS) microparticles per day. 

Innovative solutions for friendly food packaging prove the use of alternative feedstocks for plastic production compared with non-renewable plastics able to be recycled, providing antibacterial activity for a long time to prolong the shelf life of stored food [4,5], consumer safety, and adequate mechanical and thermal properties. 

In recent years, serious efforts have been made to develop sustainable food packaging materials to ensure full food quality. Biopolymers obtained from different natural resources are considered an attractive alternative to non-biodegradable petroleum-based plastics, as they are renewable, environmentally friendly, and biodegradable. Among the biodegradable polymers used to obtain food packaging, the best known are polylactic acid (PLA) and polyhydroxyalkanoates (PHAs). The use of PLA for food packaging is limited because it has low mechanical and thermal properties, low ductility, and its oxygen barrier properties are quite low compared to conventional polymers. These drawbacks are overcome by the introduction of plasticizers in the PLA matrix [6,7,8]. Polyhydroxybutyrate (PHB) and its copolymer, poly(3-hydroxybutyrate-co-3-hydroxyvalerate) (PHBV) are the two most representative natural polyesters of microbial origin belonging to the PHA family. Although PHB shows rigidity similar to PP [9], its sensorial results on the food were positive. The nanocomposites based on PHB containing graphene nanoplatelets (Gr-NPs) in a concentration of 0–1.3 wt% prepared by a casting solution [9] and silver in a single step approach [10], in addition to blends with PLA [11], have been investigated for the design of food packaging. PHVB blended with natural rubber (NR) by melt processing [12], plasticized with PEG and incorporating 13 wt.% of active compounds (carvacrol-CA, eugenol-EU) obtained by spraying [13], or loaded with *Salmonella Enteritidis* bacteriophage Felix O1, for potential use as an anti-Salmonella agent [14], indicate their potential commercial applications in safe food packaging. Overall, migration tests for the PLA/PHB (85:15) performed in non-polar and polar food simulants showed that the total amount of non-volatile substances are in the accepted legislative limit. It was found that the mechanical properties of PLA/PHB blends were enhanced by addition of the plasticizer, and the total amount of non-volatile substances migrated in contact with non-polar and polar food simulant are in the accepted legislative limit [15]. PLA/PHBV blends were used in food packaging [16], medical devices [17], and wastewater treatment [18]. The incorporation of 0.25 wt% nanocrystalline cellulose improves the morphology, mechanical, and barrier properties of PLA/PHBV composites obtained by the solvent casting method [18].

Developing antimicrobial packaging materials may prevent or delay food spoilage on the surface of the food [19,20,21,22,23,24,25,26]. An approach to the problem of microbial adhesion involves pretreating the sensitive surface with an antimicrobial agent. When impregnated with biocides or antibiotics, some materials can resist bacterial colonization as long as antibacterial agents are released from their surfaces [27]. The European Union has established that the chemicals that could migrate into the food from food packaging should not exceed 60 mg kg^−1^ of food, or 10 mg dm^−2^ relative to the surface area [28]. Many studies have reported the use of inorganic nanoparticles, such as silver (Ag) [29,30], iron oxide (Fe_3_O_4_) [31], copper oxide (CuO) [32], and zinc oxide (ZnO) [33], both as reinforcing agents for improving the mechanical properties of polymeric biocomposites as well as for adding functional properties, such as antimicrobial activity. Methods for incorporating nanoparticles, chemical composition, crystallinity, size, and shape can be controlled in order to adjust the properties of the packaging materials [34,35]. However, nanoparticles can migrate to packaged foods [36,37,38]. ZnO nanoparticles (NPs) have high absorption in the UV field and therefore are used for various applications such as photocatalysis [39], medicine, cosmetics, medical dressings [40,41], optoelectronic and medical imaging devices [42], antibacterial coatings [43], and food packaging [44,45,46,47]. In addition, ZnO nanoparticles are considered non-toxic and recognized by the FDA as safe substances, and recent studies have reported that they do not cause DNA damage to human cells [48]. According to the scientific state of the art, it is well known that the synthesis methods of ZnO NPs influence their morphology and properties [49,50] and their doping with metal ions contributes to the improvement of its properties, such as the increase of lattice defects, the generation of electron–hole pairs, and the shift of the spectral response to the visible range of spectra [51,52]. Therefore, ZnO NPs can be synthesized by precipitation [53], the hydrothermal method [54], electrochemical decomposition [55], the sono-chemical method [56], and sol-gel [57]. The precipitation method has some advantages over other physical or chemical synthesis methods, among which the need for a simple and unsophisticated apparatus and the possibility of obtaining large-scale nanoparticles with controlled shape and size. Moreover, this method allows rigorous control of nuclear and particle growth in solution [52,58,59]. ZnO doping with Fe ions effectively modifies the structural and morphological properties, as well as the electrical, optical, and magnetic properties, which can give these nanomaterials applications in different fields [60,61]. Nanocomposite films based on PLA or poly(3-hydroxybutyrate-co-3-hydroxyvalerate (PHBV) and ZnO NPs manufactured by melt compounding, with multifunctional properties (water vapor barrier, antimicrobial activity), have been reported, which recommends them for use as food packaging [43,62,63,64]. The difficulty of incorporating the inorganic antimicrobial agent in polymeric matrices by the melting or casting processes consists of its agglomeration, which need to incorporate a huge amount of antimicrobial agents, leading to toxicity increasing the migration in food simulants and deterioration of the properties in the food packaging [64,65].

Electrospinning is a versatile technique, applied to polymers to obtain nanofibers with functional properties. The principle of operation is to create a high voltage electric field between the polymer solution and the metal collector on which it is deposited in the form of fibers. Nanofibers obtained by electrospinning have improved physicochemical properties compared to fibers at the macro level and are therefore increasingly being researched for use in new food packaging systems. The advantage of nanofibers resides in the large ratio between surface area and volume, of 1–3 orders of magnitude higher compared to thin films made of the same material [66]. In addition, the electrospinning process is non-invasive and does not require the use of chemicals or high temperatures to obtain fibers. Electrospinning technology can use synthetic or natural polymer solutions, polymer mixtures, nanoparticles, and antimicrobial agents, which have a viscosity in solution suitable for producing nanofibers. For example, poly(vinyl alcohol) (PVA)-based nanofibers containing varying amounts of Fe-doped ZnO nanoparticles with antibacterial properties [31], ethyl cellulose/gelatin, and ZnO compositions with excellent hydrophobicity, water stability, and antimicrobial activity suitable for potential use in food packaging [47], chitosan nanofibers containing ZnO for medical applications [40], PHBV and ZnO NPs electrospun composite fibers for optoelectronic devices, and the biomedical imaging [42] have been reported. However, the investigated electrospun nanosystems based on PHBV and ZnO and deposited onto PLA film were not reported in terms of their morphology, migration, and antimicrobial characteristics proved by ROS generation. Antimicrobial food packaging that is sustainable, uses ecological technology for their fabrication, and does not release particles above the specific limit are required on the market.

The novelty of this paper consists of the incorporation of ZnO:Fe metal oxide nanoparticles/antimicrobial agents in PHBV through electrospinning/electrospray technology and collecting the produced nanofibers onto the PLA film. The objectives of this paper were: (i) to incorporate Fe-doped ZnO nanoparticles in PHBV and to coat the PLA film with a maximum thickness of 0.1 mm by electrospinning; (ii) to investigate the structural (FT-IR, XRD), morphology (SEM), compositional analysis (XPS, EDX), and migration in three food simulants as well as antimicrobial properties in order to assess their potential for food packaging.

## 2. Materials and Methods

### 2.1. Materials

PLA pellets Ingeo^®^ Biopolymer 4032D type (NatureWorks, Minnetonka, MN, USA) containing renewable resources is characterized by excellent optical, gas, and oil barrier properties. It is approved for use in food packaging applications. In this study, PLA was used as flexible films with the role of a coating substrate for electrospun nanofibers. PHBV pellets containing 12 mol% polyhydroxyvalerate (GoodFellow, Huntingdon, UK) show similar properties to polyolefins, a density of 1.25 g/cm^3^, an elongation at break of 35%, and tensile strength at a break of 23 MPa. PLA is very hygroscopic and might retain moisture from the air, leading to degradation of macromolecular chains, reducing product viscosity and resistance. Therefore, prior to use, the PLA and PHVB pellets were dried in an oven with air circulation at a temperature of 50 °C for 24 h (moisture content < 200 ppm). Dichloromethane and ethyl alcohol solvents and food simulants (3% acetic acid (*w*/*v*), 10% ethyl alcohol (*v*/*v*)), and 3% HNO_3_ solution were of an analytical grade.

Materials and reagents used for the preparation of ZnO NPs were: zinc nitrate- Zn(NO_3_)_2_·2H_2_O (Alpha Aesar, Bio Aqua Group, Targu Mures, Romania), iron nitrate nonahidrate- Fe(NO_3_)_2_·9H_2_O (Alpha Aesar, Bio Aqua Group, Targu Mures, Romania), absolute ethanol C_2_H_5_OH-EtOH (Merck, Redox Life Tech, Cluj-Napoca, Romania), and sodium hydroxide (NaOH) (Alpha Aesar, Bio Aqua Group, Targu Mures, Romania). All chemicals were of analytical grade and used without further purification. The aqueous solutions were prepared with Milli-Q water obtained from Direct-Q 3UV system (Millipore, Bedford, MA, USA).

### 2.2. Synthesis of Fe-Doped ZnO Nanoparticles

Fe doped ZnO NPs—ZnO:Fex (x = 0%, 0.3%, 0.5%, 0.7%, 1.0%) were synthesized by chemical precipitation. The ZnO:Fe nanoparticles were obtained according to literature data [67] with minor changes necessary for the purposes of this work.

In a typical procedure, stoichiometric amounts of Zn(NO_3_)_2_·6H_2_O (98%) and Fe(NO_3_)_2_·9H_2_O were dissolved in 100 mL of ultrapure water and mixed together to form a homogeneous solution. Subsequently, a solution of 2 M NaOH was added dropwise at a constant stirring rate until a white precipitate of zinc hydroxide was obtained. After pH = 12 was reached, the mixture was continuously stirred for 4 h at room temperature. The obtained ZnO:Fex was washed with ultrapure water and then dried at 65 °C for 24 h.

### 2.3. Processing of PLA Film

Homogenous PLA films with dimensions 150 × 150 × 0.1 mm were obtained by hot-pressed on a laboratory press under the following conditions: preheating for 5 min at 150 bar and pressing for 2 min, at a pressure of 150 bar, both at 190 °C, followed by cooling for 45 min and 150 bar.

### 2.4. Coating of PLA Films with PHBV/ZnO:Fe Electrospun Nanosystems

First, 8 wt% PHBV solution was prepared by dissolving PHBV pellets in a mixture of dichloromethane and ethanol at a vol. ratio of 6:1 between solvents by using a laboratory mixer at a temperature of 60 °C and a speed mixing of 400 rpm, for 2 h. After obtaining a homogeneous solution, 1 wt% of Fe-doped ZnO antimicrobial agent containing: 0 wt%, 0.3 wt%, 0.5 wt%, 0.7 wt%, and 1 wt%, respectively, of Fe was incorporated in the prepared PHBV solution. The resulted solutions coded PHBV:ZnO:Fe0, PHBV:ZnO:Fe0.3, PHBV:ZnO:Fe0.5, PHBV:ZnO:Fe0.7, and PHBV:ZnO:Fe1 were introduced into a syringe pump, having as a pin a pointless stainless steel needle, with an internal diameter of 0.168 mm. Nanofibers based on PHBV and 1 wt% ZnO:Fex NPs with different content of Fe were deposited onto PLA films by the electrospinning process using an uniaxial electrospinning equipment (TL Pro-BM, Tong Li Tech Co., Ltd., Bao An, Shenzhen, China), at a voltage in the range of 16.3–16.7 kV, a flow rate between 0.4 to 3.6 mL/h and 14 cm being the distance between the needle to cylindrical disc collector. The electrospinning process was performed in an environment under temperature of 28.5 ± 1.5 °C and relative humidity of 28 ± 1%. Nanofibers of PHBV deposited onto PLA film and PLA films were used for comparative purposes.

### 2.5. Investigation Methods

#### 2.5.1. Particle Size Distribution

The size measurements of Fe-doped ZnO nanoparticles were performed by a dynamic light scattering (DLS) technique (Zetasizer Nano ZSP, Malvern Instruments, Malvern, UK), and the scattered light was collected at 173°, with a red laser wavelength of 632.8 nm (He/Ne). In addition, 0.1 g of each doped ZnO were immersed in 5 mL of ultrapure water and sonicated for 5 min. Then, 3 suspension drops were dispersed into 10 mL solution 1 mM NaCl, homogenized again and analyzed by using a 12 mm cell (DTS 0012). Measurements of Z average, size, and polydispersity index were performed in triplicate, and the results are expressed as mean values ± standard deviation.

#### 2.5.2. Scanning Electron Microscopy (SEM)

SEM analysis was done using Hitachi SU-8230 (Tokyo, Japan) with a cold field emission gun at 30 kV. Before the analysis, all samples were sputter coated with 10 nm Au in Ar atmosphere. Energy dispersive X-ray analysis (EDX) was done using an Oxford Instruments detector (Oxford, UK) and AZtech software (Greeley, CO, USA).

#### 2.5.3. Structural Analysis by X-ray Diffraction

The structural characterization and crystalinity degree for PLA/PHBV/ZnO:Fex samples were also investigated by a Bruker D8 Advance Diffractometer (Rheinstetten, Baden-Württemberg, Germany) in Bragg Brentano geometry equipped with Ge(111) in the incident beam, a fast LynxEye detector, and Cu–Kα1 radiation (λ = 1.54060 Å), (40 kV, 40 mA) over the range of 2θ = 3–70°, at ambient temperature. Before being embedded into PHBV, ZnO:Fex nanoparticles were structurally characterized. The crystallite dimensions were determined with MATCH software (Kreuzherrenstr, Germany).

#### 2.5.4. Fourier Transform Infrared Spectroscopy (FT-IR)

The PHBV/ZnO:Fex nanosystems were analyzed in reflection mode, at room temperature, by means of an Atenuated Total Reflectance—FT-IT (INTERSPEC 200-X Spectrophotometer, Interspectrum, Tartumaa, Estonia) having a ZnSe crystal with an incidence angle of 45°. All spectra were acquired in a wavenumber range from 3500 to 750 cm^−1^, at 4 cm^−1^ resolution, as the average of 20 scans, using air as background. PLA film and PHBV nanofibers deposited onto PLA film were used for comparative purposes.

#### 2.5.5. X-ray Photoelectron Spectroscopy (XPS)

X-ray photoelectron spectroscopy (XPS) analysis was performed with a SPECS custom-build (Berlin, Germany) using Al Kα X-rays (1486.61 eV). Each sample showed several Ar ions etchings until the XPS spectra remained unchanged in shape and intensity. At this stage, the XPS spectra reflect the real composition of samples. In order to avoid artificial reduction of different oxidation stats of elements, the etching was performed by using Ar ions accelerated at a maximum 1000 V voltage with a filament current of 10 mA. XPS spectrum analysis was performed with CasaXPS software. Spectrum calibration was done considering the 284.6 eV C 1s line associated with C–C or C–H bindings.

#### 2.5.6. Migration Tests

Specific migration tests were performed in ethanol 10% (*v*/*v*) (food simulant A) and acetic acid 3% (*w*/*w*) (food simulant type B) and by using sample ashes treated with 3% nitric acid (HNO_3_) solution. Two-faced migration was performed by immersing circular specimens of 3.14 cm^2^ total area of each sample in plastic vials containing 20 mL of food simulants to keep the relation 6 dm^2^ kg^−1^ as it is indicated in the current legislation [28]. The samples were stored in a controlled atmosphere at 70 °C during 2 h. Blank simulants were also prepared and analyzed in each case.

After the migration tests, films were removed and simulants were analyzed for ZnO and Fe released by Inductive Coupled Plasma Mass Spectrometry detection (ICP-MS Agilent 7500a, Palo Alto, CA, USA). In addition, the content of inorganic material was determined by testing the ash resulting from the calcination of the samples at a temperature of 550 °C for 1.30 h. The ash was suspended in approximately 50 mL of 3% HNO_3_ solution (*v*/*v*). The samples were stored in the refrigerator until the analysis. All samples were tested in triplicate.

The detection was performed using the following conditions: 5 repetitions of each mass; full-spectrum mode, acquisition time was 0.3 s per mas; and peristaltic pump aspiration speed was set at 0.3 revolutions per second.

Calibration curves were prepared with standard solutions of Zn and Fe in each simulant as well as in 3% nitric acid. The following concentration ranges were used in case of Fe calibration curves: 20–1000 ng/mL (3% acetic acid), 116–1000 ng/mL (10% ethanol), and 10–1000 ng/mL (3% nitric acid). In the case of Zn, the calibration curves covered the concentration ranges: 10–1000 ng/mL (3% acetic acid), 27–1000 ng/mL (10% ethanol), and 3–1000 ng/mL (3% nitric acid). The limits of detection (LOD) and quantification (LOQ) were calculated as the average signal of blank plus three (LOD) and ten (LOQ) times its standard deviation.

#### 2.5.7. Bacterial Adherence

*Pseudomonas aeruginosa* (ATCC-27853) was used for this analysis. The tested microorganism was obtained from Food Biotechnology Laboratory, Life Sciences Institute, University of Agricultural Sciences and Veterinary Medicine Cluj Napoca, Romania. The bacteria were cultured on Muller–Hinton Agar and cultures were stored at 4 °C and sub-cultured once a month. The bacterial adherence was tested to simulate the affinity of microorganisms to the implant surface, directly after implantation. A protocol adapted from Tanner et al. [68] was applied for the *P. aeruginosa* on both uncoated and coated specimens. The bacteria were precultured from a frozen glycerol preparation and inoculated in 45 mL Tryptic Soy Broth for 16 h at 37 °C. After harvesting the bacteria by centrifugation (4000 rpm, +4 °C, 10 min), they were washed once with a physiological sterile saline solution. Then, cells were re-suspended in physiological saline solution at a concentration of ~0.035%, and the absorbance was measured at 550 nm, which corresponded to ~1 × 10^7^ colony-forming units (CFU). The suspension was gently sonicated and vortexed to homogenize the solution. Then, the specimens were placed in 15 mL test tubes with 5 mL of the bacterial suspension. After 30 min at room temperature, the specimens were washed three times in abundant physiological saline solution and gently dried without touching the surface. Thereafter, the bacterial samples from the specimen surfaces were collected for analysis of viability with micro brushes into 2 mL microtubes containing 900 μL of Tryptic Soy Broth with 10% glycerol. Then, the bacteria were homogenized, serially diluted in physiological saline solution (10 μL of 1:10, 1:100, and 1:1000), and cultured on Muller–Hinton agar plates. CFU measurements were done after 24 h of culturing at 37 °C.

#### 2.5.8. Evaluation of the Reactive Oxygen Species (ROS) Generation

By using the Electron Spin Resonance (ESR) also known as Electron Paramagnetic Resonance (EPR) coupled with the spin-trapping probe technique, the ROS production of PLA/PHBV/ZnO:Fex in DMSO (dimethyl sulfoxide) suspensions was monitored. In addition, 5,5-dimethyl-1-pyrroline *N*-oxide (DMPO, Sigma-Aldrich, Merck, KGaA, Darmstadt, Germany) was used as a spin trapping agent. The samples were investigated by dispersing 10 mg of PLA/PHBV/ZnO:Fex in 1 mL of DMSO. These suspensions were homogenized in an ultra-sound bath (30 s) before use. The DMPO concentration was 0.2 M. The PLA/PHBV/ZnO:Fe 0.3% sample was transferred into the quartz cell of spectrophotometer optimized for liquid measurements, and the experimental spectra were recorded. The measurements were recorded with a Bruker E-500 ELEXSYS X-band (9.52 GHz) spectrometer (Rheinstetten, Baden-Württemberg, Germany) in the same experimental conditions.

#### 2.5.9. Statistical Analysis

All data were expressed as the mean ± standard deviation of the mean. Statistical analysis was performed by using GraphPad Prism 8 software.

## 3. Results and Discussion

### 3.1. Dimension Size Measurement

**Figure 1:**
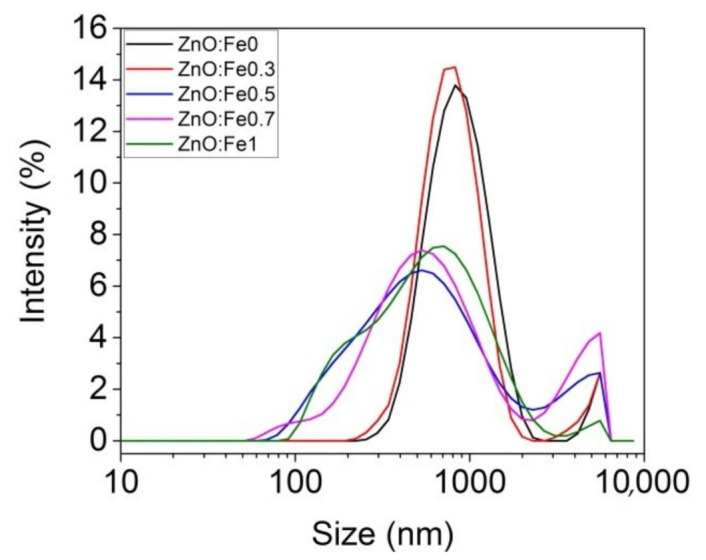
Size distribution of ZnO:Fex nanoparticles.

The analysis of the dimensional distribution and size of the prepared ZnO:Fex nanoparticles was determined by the spectroscopic correlation of the photons by the laser diffusion technique (DLS) as can be seen in Figure 1 and Table 1.

Table 1 shows that the average Z decreased with the increase of Fe in ZnO NPs. ZnO:Fex nanoparticles are polydispersed (polydispersity index (Pdi) < 0.4) and showed two peaks with different intensities in the band ranging from 608 nm to 900 nm (>86% distribution) and from 4143 nm to 5280 nm (<13% distribution), respectively (Table 1).

### 3.2. Scanning Electron Microscopy/Energy Dispersive X-ray (SEM/EDX) Analysis

**Figure 2:**
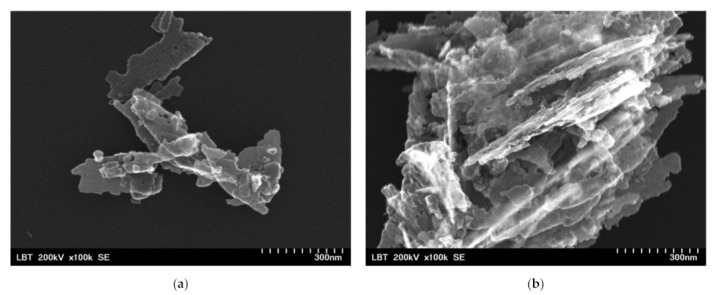
SEM images for ZnO:Fex nanostructures: (**a**) ZnO:Fe0 and (**b**) ZnO:Fe0.7.

**Figure 3:**
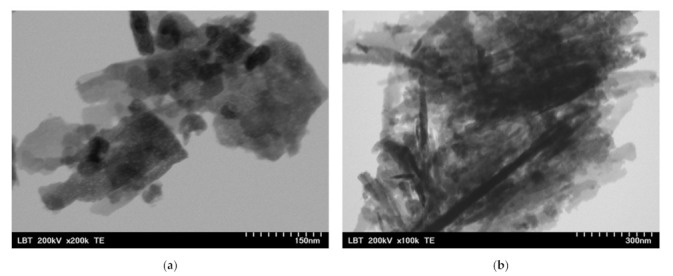
TEM images for ZnO:Fex nanostructures: (**a**) ZnO:Fe0 and (**b**) ZnO:Fe0.7.

Before the incorporation in the prepared PHBV solution, the ZnO:Fex nanoparticles were investigated by TEM and SEM. As an example, TEM and SEM images of pure ZnO and ZnO:Fe 0.7% were recorded and shown in Figure 2 and Figure 3, respectively.

The images show that ZnO and ZnO:Fe0.7% nanoparticles have a aggregated “sheets” like morphology with different lengths and sizes [69].

**Figure 4:**
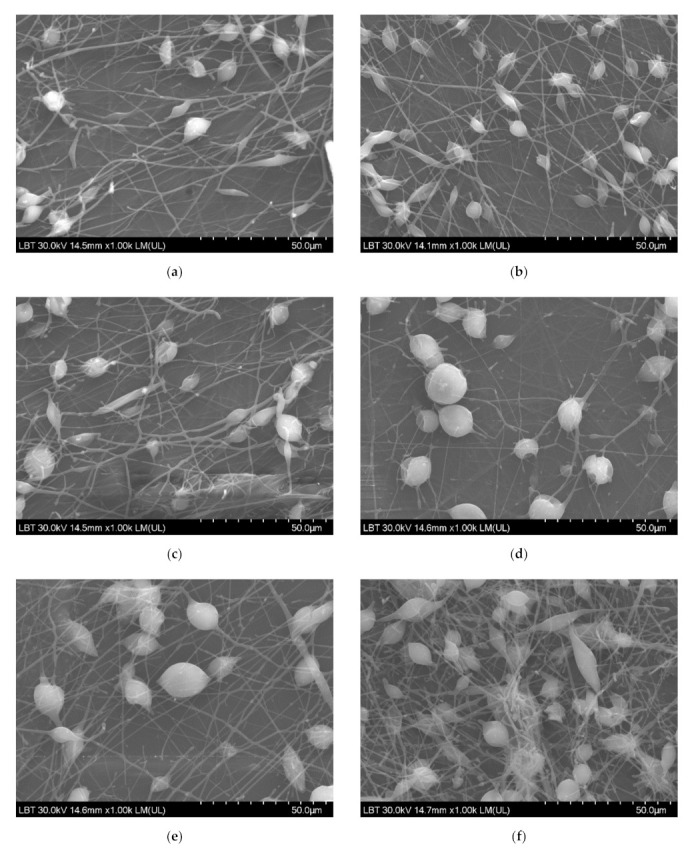
SEM images for PLA/PHBV/ZnO:Fex nanostructures: (**a**) PLA/PHBV, (**b**) PLA/PHBV/ZnO:Fe0, (**c**) PLA/PHBV/ZnO:Fe0.3, (**d**) PLA/PHBV/ZnO:Fe0.5, (**e**) PLA/PHBV/ZnO:Fe0.7, and (**f**) PLA/PHBV/ZnO:Fe1.

SEM micrographs revealed a uniformly distributed PHBV containing ZnO:Fex nanostructures with “beads” morphology (Figure 4). The presence of random PLA/PHBV/ZnO:Fex clusters was also noticed, most likely from the electrospinning solution.

Energy Dispersive X-ray Analysis (EDX) allowed the elemental composition of PLA/PHBV/ZnO:Fex nanostructures. The elemental compositions are presented in Table 2. EDX analysis revealed the PLA/PHBV/ZnO:Fex elemental composition: C (~59 wt%), Zn (increasing concentrations 0.1–1 wt%), O (~40 wt%) as main constituents and Cu from the sample support material. Fe concentration was under the detection limit of the analysis.

**Figure 5:**
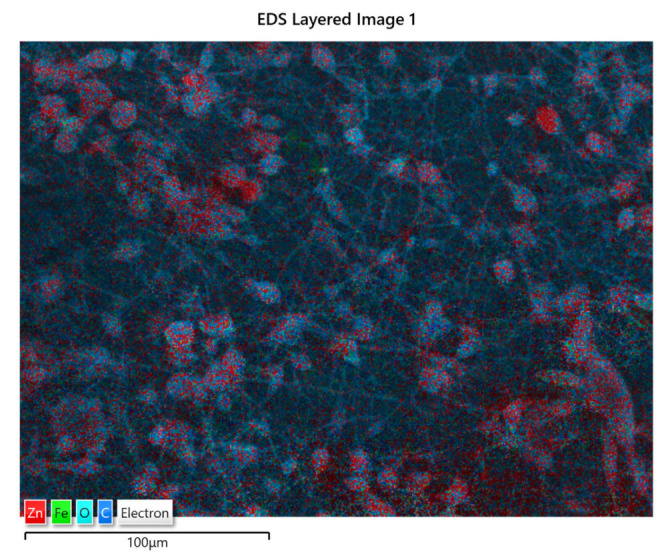

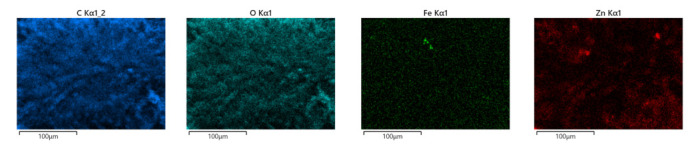
EDS mapping of PLA/PHBV/ZnO:Fe1%.

The distribution of ZnO:Fe nanoparticles into the polymer matrix was analyzed by the EDS mapping. As an example, the EDS mapping of PLA/PHBV/ZnO:Fe1% is shown in Figure 5. The mapping indicates the presence of ZnO:Fe nanoparticles within both spheroidal structures and the fibers which connect them. The spheroids are formed around bundles of ZnO:Fe nanoparticles resulting from a non-uniform dispersion in the PHBV solution. Here, ZnO nanoparticles tend to agglomerate as long as their dispersion in the polymer matrix is not stabilized.

### 3.3. X-ray Diffraction

Structural characterization and crystallinity degree of PLA/PHBV/ZnO:Fex samples were investigated by X-ray Diffraction (XRD).

**Figure 6:**
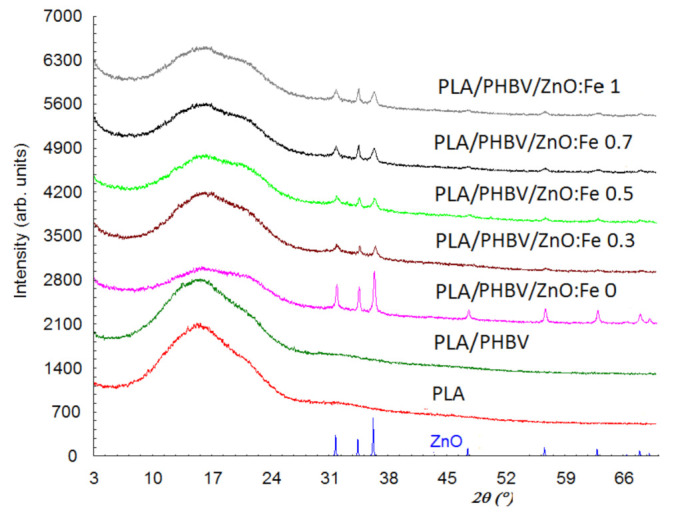
X-ray diffraction patterns of PLA/PHBV/ZnO:Fex samples compared with ZnO NPs, PLA film, and PHBV nanofibers.

Figure 6 shows the diffraction patterns of ZnO, PLA, PLA/PHBV, and PLA/PHBV/ZnO:Fex composite samples.

X-ray diffraction patterns for the PLA and PHBV samples show that they have only one amorphous phase, while the other samples show both the amorphous and crystalline phase due to the ZnO incorporation. The diffractogram for the amorphous phase shows two diffraction halos—one of them with a maximum at about 2θ = 16°, and the other halo with lower intensity and shielded by the diffraction lines of ZnO is found at about 32°. In addition, the first diffraction halo at 2θ = 16° seems to be split. The interplanar distance (d) corresponding to 2θ = 16° is 5.6 Å and, for 2θ = 32°, it is 2.8 Å. This suggests that the PLA are stratified, 5.6 Å being the distance between layers and the diffraction angles corresponding for the two halos are the order 1 and order 2 of diffraction from the Bragg relationship. The diffraction peaks at 2θ: 31.75, 34.40, 36.24, 47.51, 56.60, 62.80, and 67.90° in PLA/PHBV/ZnO:Fex samples indicate the presence of the ZnO crystalline phase. Although the amount of ZnO in samples has a maximum of 1%, the diffraction maxima are prominent since the ZnO phase is evenly distributed on the sample surface and the diffraction information is collected from the surface (usually 0.1 mm). The degree of crystallinity, Xc, was evaluated as the ratio of the diffraction peaks’ area and the total diffraction area, which includes diffraction peaks and amorphous halo [70]. To assess the degree of crystallinity, the Reflex computer program part of the Material Studio software suite was used [71]. The values obtained for Xc are summarized in Table 3. The degree of crystallinity is 100% for ZnO nanoparticles and, for PLA and PHBV, is close to 0%.

In the case of PHBV/ZnO electrospun nanosystems used to coat PLA films, the doping with Fe decreases the size of these crystallites ranging from 13 nm for ZnO to 11.5 nm for ZnO: Fe 0.3%.

### 3.4. ATR-FT-IR Analysis

FT-IR analysis was performed on PLA/PHBV/ZnO:Fex electrospun samples compared with PLA film to identify the effect of ZnO:Fex on the chemical structure of nanosystems.

**Figure 7:**
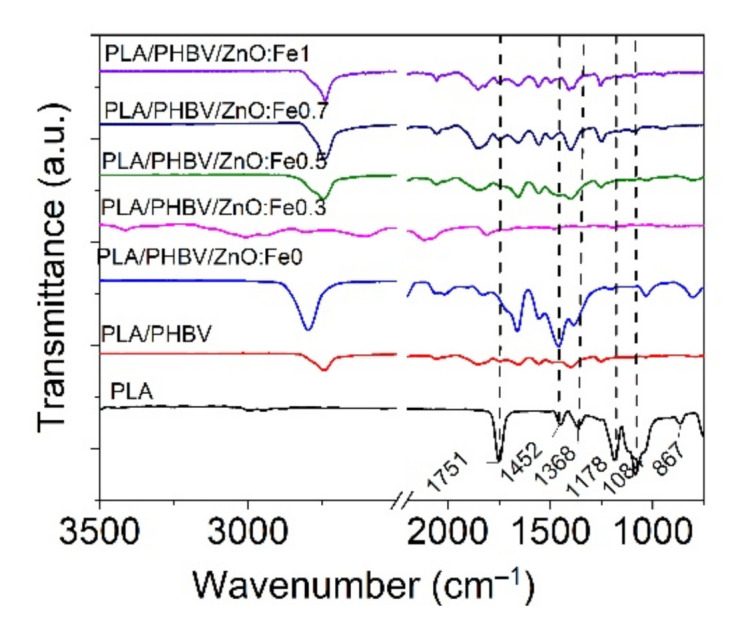
Normalized FT-IR spectra for PLA/PHBV/ZnO:Fex electrospun nanosystems compared with those for PLA film and PHBV nanofibers in the range of 3500–750 cm^−1^.

The PLA film spectrum showed two small absorption peaks located at 2992 cm^−1^ and 2947 cm^−1^ (symmetric and asymmetric C–H stretching vibrations), a strong and sharp absorption peak at 1751 cm^−1^ (C=O stretching of the carbonyl group), 1452 cm^−1^ and 1368 cm^−1^ (C–C stretching vibrations), 1084 cm^−1^ (rocking vibrations of CH_2_ bond), 867 cm^−1^ and 751 cm^−1^ (amorphous and crystalline phases) as can be seen in Figure 7 [42,72]. The deposition of PHBV onto PLA film did not reveal an absorption peak at around 1720 cm^−1^ assigned to the vibration of crystalline stretching of C=O carbonyl group in PHBV [73,74] because PLA and PHBV are incompatible polyesters. It is observed from Figure 7 that the incorporation of ZnO:Fe nanoparticles into the PHBV matrix revealed a prominent peak located at around 2790 cm^−1^, implying that there were hydrogen bond interactions between the vibrations of the CH_2_ group of PHBV and the hydroxyl group of the nanoparticles [44].

### 3.5. Qualitative Analysis by XPS

**Figure 8:**
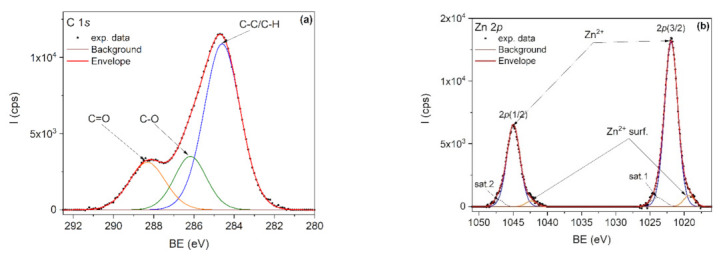
XPS spectra of PLA/PHBV/ZnO:Fe0.3 sample: (**a**) C 1s and (**b**) Zn 2p.

The elemental composition of the nanocomposites was investigated by XPS. It is known that PLA degrades under X-ray radiation [75] and therefore the C1s spectrum was first recorded, followed by Zn 2p and O1s spectra. For example, Figure 8a,b showed the C1s and Zn 2p core-level spectra, corresponding to the PLA/PHBV/ZnO:Fe0.3 sample. The deconvolution of the C 1s core level (Figure 5) was done by taking into account the specific lines for PLA and PHBV: C–C/C–H, C–O and C=O. PLA is expected to show equal proportions of the components, while PHBV is expected to show a larger C–C/C–H component. The C–C/C–H component is bigger than expected due to the adventitious carbon contamination of the sample.

In the deconvolution of the Zn 2p doublet spectrum (Figure 8b), the 2p(3/2) and 2p(1/2) peaks positioned at 1021.9 and 1044.9 eV are attributed to Zn^2+^ from the ZnO lattice. The doublet positioned at lower binding energies, namely 1019.3 eV for 2p(3/2) and 1042.3 eV for 2p(1/2), is attributed to Zn^2+^ surface states. Two shakeup satellites positioned at 1023.9 eV and 1047.1 eV were also used in spectra deconvolution.

### 3.6. Migration Tests

An ICP-MS was employed to quantify the Zn and Fe ions in PHBV/ZnO:Fex electrospun nanosystems. The LOD of the specific migration in 3% acetic acid simulant, 10% ethanol simulant, and 3% nitric acid were: 6 ng/mL, 35 ng/mL, and 3 ng/mL, respectively, for Fe and 3 ng/mL, 8 ng/mL and 1 ng/mL, respectively, for Zn—while the LOQ of the specific migration in 3% acetic acid simulant, 10% ethanol simulant, and 3% nitric acid were: 20 ng/mL, 116 ng/mL, and 10 ng/mL, respectively, for Fe, and 10 ng/mL, 27 ng/mL, and 3 ng/mL, respectively, for Zn.

According to Annex II of EU Commission Regulation 10/2011, the food packaging materials must not release substances in quantities exceeding the specific migration limits for Fe = 48 mg/kg food or food simulant and Zn = 5 mg/kg food or food simulant.

Table 4 shows that the results of specific migrations for Zn and Fe from PLA-based compositions coated with PHBV and Fe-doped ZnO nanostructures performed using food simulants A and B and also after the treatment of ash with 3% (*v*/*v*) HNO_3_ do not exceed the limits specified by the current legislation. Moreover, some values are below the detection limit of the analytical method. In all the cases, the results are below the overall migration limit of 60 mg/kg of the food simulant as well as those specified for Zn and Fe in Annex II of the Regulation (EU) No. 10/2011. Among the three migration tests performed, it can be found that the Fe migration test in HNO_3_ solution is the most severe, while the 3% acetic acid solution is the most severe for Zn migration. Similar results were reported by Vasile et al. [63] in the case of testing the specific migration of Zn and Cu ions from plasticized PLA samples with embedded Cu-doped ZnO powder functionalized with Ag nanoparticles’ composites. The authors found that the migration of Zn was more sensitive that than of Cu both in acetic acid and ethanol food simulants [63]. This behavior can be explained by the strong ionization tendency of Zn [76].

### 3.7. Antimicrobial Evaluation

In the adherence test, the interaction between the cell surface and the material surface was measured. Thus, fewer *P.* *aeruginosa* cells were found attached to the PLA/PHBV/ZnO:Fex samples, as is shown in Table 5. Similar prominent bactericidal activity of polyvinyl alcohol nanofibers was reported with the increase in Fe-doped ZnO nanoparticles (NPs) concentration against Gram-positive *Staphylococcus aureus* and Gram-negative *Escherichia coli* bacterial strains [31].

The results highlight the importance of using ZnO:Fex nanoparticles on PLA matrices because all the samples that include this compound in their composition, at different concentrations, showed a lower degree of initial bacterial adhesion to their surface. The materials tested in this study succeed in inhibiting bacterial adhesions.

Generally, the antibacterial effect of ZnO NPs is due to the electronic charge, the small size, and the large surface–volume ratio that allows interactions with bacteria [77], and their capacity to generate reactive oxygen species (ROS), such as hydroxyl radicals (OH^−^), superoxide anion (O_2_^−^), and perhydroxyl (OOH) [78]. The ROS species are generated by the interaction between electrons or hole generated by the nanoparticles with the water or oxygen from the atmosphere [79]. Fe dopant ions also facilitate ROS generation having a strong toxic effect on the bacteria due to their high reactivity and strong oxidizing properties [80]. By doping, energy levels associated with the dopant are introduced into the ZnO conduction band which can facilitate the electron–hole separation increasing the probability of ROS generation. At a high doping level, the excessive amounts of doped Fe may act as the recombination centers for the electron–hole pairs by their interaction resulting in low ROS generation and consequently lower antibacterial activity [67].

### 3.8. The Evaluation of ROS Generation

The ROS generated in the presence of PLA/PHBV/ZnO:Fe0.3 nanostructure were monitored by electron spin resonance (ESR) spectroscopy coupled with a spin-trapping technique. The ROS species are involved in the mechanism of antibacterial activity of semiconductors [81].

**Figure 9:**
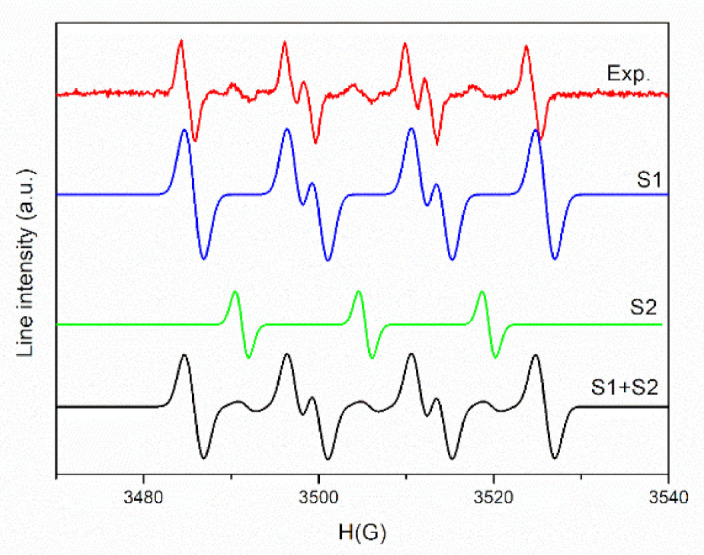
The experimental and simulated spectra of PLA/PHBV/ZnO:Fe0.3% in DMSO suspension in the presence of DMPO.

To ensure the origin of these signals, a simulation was performed. The experimental and simulated spectra of PLA/PHBV/ZnO:Fe0.3% in DMSO suspension in the presence of DMPO are shown in Figure 9.

As seen in Figure 9, a complex spectrum was obtained, which after simulation reveals the presence of two spin adducts: DMPO–OOH (S1) with hyperfine coupling constants, a_N_ = 14.2 G, a_H_ = 11.7 G, a_H_ = 1 G (relative concentration 90%) and a nitroxide-like radical (S2) (a_N_ =14 G, relative concentration 10%). The mechanism of (˙OOH) radical formation comprises two steps. In the first step, the electrons generated by the interaction of the sample with light (or photogenerated electrons) interact with absorbed O_2_ and form (˙O_2_^−^) radicals. In the second stage, a perhydroxyl (˙OOH) radical is formed by the (˙O_2_^−^) protonation reaction [82]. The presence of nitroxide-like radical is due to the N–C bond cleavage followed by the ring-opening of spin trapper (DMPO) [83].

## 4. Conclusions

Coatings of PLA film with PHBV/ZnO:Fex nanosystems were obtained by using the electrospinning process. The process of getting the antimicrobial nanosystems is simple, versatile, and uses reduced amounts of Fe-doped ZnO nanoparticles. It was conducted at room temperature, without high energy consumption, and without solvents with toxic potential. The samples showed “beads” morphology. Migration in food simulants is within the limits imposed by current legislation. From all investigated samples, the PLA/PHBV/ZnO:Fe0.3 electrospun nanosystem showed a remarkable antimicrobial effect against *P. aeruginosa* (ATCC-27853) bacterial strain due to the generation of a larger amount of perhydroxyl (˙OOH) radicals, as evidenced by using the EPR spectroscopy coupled with the spin-trapping method. XPS showed the specific C 1s core-level lines of PLA and PHBV and also the Zn 2p core-level lines for Zn^2+^ oxidation state specific to ZnO.

## 5. Patents

M. Stefan, M. Rapa, O. Pana, D. Vodnar, E. Matei, D. G. Barta, A. Popa, D. Toloman, C. Leostean, and S. Macavei, Nanostructures based on PHBV and Fe doped ZnO nanostructures and their obtaining process, Patent Request Nr. A 00322/09.06.2020.

## Figures and Tables

**Table 1:** Zetasizer parameters for Fe-doped ZnO nanoparticles determined from DLS analysis.

Fe-Doped ZnO	Average Z (nm)	Size		Intensity		Pdi
		Peak 1 (nm)	Peak 2 (nm)	Peak 1 (%)	Peak 2 (%)	
ZnO:Fe0	780.1	900.2 ± 1.5	5280 ± 126	95.8	4.2	0.347
ZnO:Fe0.3	818.6	805.4 ± 48.8	5201 ± 387	94.7	5.3	0.338
ZnO:Fe0.5	460.0	608.8 ± 76.8	4143 ± 653	86.8	13.3	0.456
ZnO:Fe0.7	443.0	670.0 ± 0.3	5021 ± 0	98.0	1.7	0.388
ZnO:Fe1	444.5	685.3 ± 21.6	4882 ± 197	98.1	1.9	0.384

**Table 2:** Elemental chemical composition of PLA/PHBV/ZnO:Fex nanostructures determined from EDX analysis.

Nanostructures	C (wt% ± 2σ *)	O (wt% ± 2σ)	Cu (wt% ± σ)	Zn (wt% ± σ)
PLA/PHBV	59.1 ± 0.3	40.8 ± 0.3	0.1 ± 0.01	0
PLA/PHBV/ZnO:Fe0	59.0 ± 0.3	40.0 ± 0.3	0.1 ± 0.01	1.0 ± 0.01
PLA/PHBV/ZnO:Fe0.3	56.9 ± 0.3	42.5 ± 0.3	0.2 ± 0.01	0.7 ± 0.01
PLA/PHBV/ZnO:Fe0.5	58.7 ± 0.3	40.5 ± 0.3	0.2 ± 0.01	0.5 ± 0.01
PLA/PHBV/ZnO:Fe0.7	59.0 ± 0.3	40.4 ± 0.3	0.3 ± 0.01	0.2 ± 0.01
PLA/PHBV/ZnO:Fe1	60.0 ± 0.3	39.5 ± 0.3	0.3 ± 0.01	0.1 ± 0.01

* (±2σ) wt% calculated automatically within the software, should not be taken as absolute measurement of *precision*.

**Table 3:** The degree of crystallinity (Xc) for PLA/PHBV/ZnO:Fex samples.

Sample	Xc (%)
PLA/PHBV/ZnO:Fe0	8.34
PLA/PHBV/ZnO:Fe0.3	8.43
PLA/PHBV/ZnO:Fe0.5	10.16
PLA/PHBV/ZnO:Fe0.7	10.44
PLA/PHBV/ZnO:Fe1	8.42

**Table 4:** Specific migration of Zn and Fe in 3% (*wt*/*v*) acetic acid, 10% (*v*/*v*) ethanol, and, after the treatment of ash with 3% (*v*/*v*) HNO_3_, 2 h at 70 °C.

Sample	3% (*wt*/*v*) Acetic Acid		10% (*v*/*v*) Ethanol		Ash Treated with 3% (*v*/*v*) HNO_3_		Theoretical Content	
	Zn, mg/kg	Fe, mg/kg	Zn, mg/kg	Fe, mg/kg	Zn, mg/kg	Fe, mg/kg	Zn, mg/kg	Fe, mg/kg
PLA/PHBV/ZnO:Fe0	2.917	<LOD	0.233	<LOD	<LOD	0.538	16.00	0
PLA/PHBV/ZnO:Fe0.3	2.722	<LOD	0.700	<LOD	<LOD	0.534	9.97	0.03
PLA/PHBV/ZnO:Fe0.5	1.830	0.022	0.048	<LOD	<LOD	0.727	9.95	0.05
PLA/PHBV/ZnO:Fe0.7	1.009	0.026	0.038	<LOD	<LOD	1.143	9.93	0.07
PLA/PHBV/ZnO:Fe1	0.796	0.035	<LOD	<LOD	<LOD	1.423	9.90	0.10

**Table 5:** Cell number (CFU) at different successive dilution for ZnO:Fe samples.

Sample	1:10	1:100	1:1000
PLA	33	2	0
PLA/PHVB	10	7	1
PLA/PHBV/ZnO:Fe0	3	3	0
PLA/PHBV/ZnO:Fe0.3%	0	0	0
PLA/PHBV/ZnO:Fe0.7%	7	5	4
PLA/PHBV/ZnO:Fe1%	11	8	4

## Data Availability

The data presented in this study are available upon request from the corresponding author.
